# Association between Obstructive Sleep Apnea and Chronic Kidney Disease According to Sex, Long Working Hours: The Korean National Health and Nutrition Examination Survey (2019–2020)

**DOI:** 10.3390/life13081625

**Published:** 2023-07-26

**Authors:** Sung-Min Jung, Mee-Ri Lee

**Affiliations:** 1 Department of Surgery, Inje University, Ilsan Paik Hospital, Goyang-si 10380, Republic of Korea; sungmin@paik.ac.kr; 2 Department of Preventive Medicine, Soonchunhyang University College of Medicine, Cheonan-si 31151, Republic of Korea

**Keywords:** chronic kidney disease, sex characteristics, obstructive sleep apnea, overtime work, STOP-BANG questionnaire

## Abstract

This study aimed to investigate whether obstructive sleep apnea (OSA) is associated with an increased risk of chronic kidney disease (CKD) and to perform subgroup analysis by sex and working hours. This cross-sectional study was conducted on 8157 subjects who participated in the Korea National Health and Nutrition Examination Survey (KNHANES). The adults completed the STOP-BANG score to measure their risk of OSA, and blood and urine samples were collected to ascertain the severity of CKD based on the estimated glomerular filtration rate and urine albumin-to-creatinine ratio. Multivariate logistic regression was used for complex sample analysis. After fully adjusting for the confounding variables, the high-risk OSA group showed a significantly higher risk of developing albuminuria and CKD than the low-risk group, particularly among men. Odds ratio (OR) 1.72, 95% confidence interval (CI) 1.13–2.6 and (OR 1.67, 95% CI 1.14–2.45), respectively. Additionally, men who worked for 40 h/week showed a significant association between OSA, CKD, and albuminuria. This study supports the link between OSA and the risk of kidney disease, especially among men and those who work long hours. Screening and treating OSA may be a crucial strategy for preventing kidney disease, particularly in high-risk populations.

## 1. Introduction

Obstructive sleep apnea (OSA) is a condition characterized by repetitive episodes of partial or complete airway obstruction during sleep, leading to fragmented sleep and decreased oxygenation [1]. Global estimates using five or more events per hour suggest that there are 936 million people aged 30 to 69 years with mild to severe OSA worldwide and 425 million people with moderate to severe OSA worldwide [2]. The estimated prevalence in North America is approximately 20 to 30% in men and 10 to 15% in women [3]. However, it is important to note that OSA is often undiagnosed, so the actual number of people affected may be higher than the estimated figures [4]. OSA has a range of negative effects on health including type 2 diabetes, cardiovascular disease, and all-cause mortality in the elderly [5].

The STOP-BANG questionnaire is a quick and easy screening tool used to assess a person’s risk of having OSA [6]. Additionally, the questionnaire is less time-consuming and less expensive than laboratory polysomnography (PSG), which is the golden standard diagnostic tool to confirm the presence of OSA. In the population studied, the STOP-Bang score had a sensitivity of 83.6% for detecting OSA with an apnea-hypopnea index (AHI) of greater than five events per hour of sleep [7].

Globally, chronic kidney disease (CKD) has a major effect on global health. For example, 1.2 million people die from CKD, and the all-cause mortality rate increased by 41.5% between 1990 and 2017 [8]. Studies have shown that individuals with OSA are more likely to develop chronic kidney disease and end-stage renal disease than those without OSA [9]. Systemic reviews for 18 articles found that patients who received a diagnosis of OSA demonstrated a significantly increased odds ratio (OR) for experiencing a poorer renal outcome, elevated levels of albuminuria/proteinuria, and a decreased estimated glomerular filtration rate [10]. Another systematic review of six cross-sectional and three retrospective studies suggested a significant association between OSA and CKD, particularly in the context of more severe presentations [11]. However, based on the meta-analysis findings, continuous positive airway pressure treatment for OSA does not have a clinically significant impact on the estimated glomerular filtration rate [12].

The OSA patterns differ between men and women. Generally, women tend to experience mild OSA during non-rapid eye movement sleep, resulting in a lower AHI than men [13]. However, OSA during rapid eye movement, sleep is more prevalent in women [13]. While men have a higher risk of CKD progression [14], previous studies have not fully explored the sex differences in the association between OSA and CKD.

Although a few studies have suggested a relationship between long working hours and CKD development [15,16], none have examined how working hours affect the association between OSA and CKD to the best of our knowledge.

Therefore, our study aims to investigate sex-based differences in the association between OSA and albuminuria/CKD, while also performing a subgroup analysis using working hours.

## 2. Materials and Methods

### 2.1. Study Design

This study used data from the Korean National Health and Nutrition Examination Survey (KNHANES) VIII (2019–2020). KNHANES is an ongoing surveillance system that was established with the primary aim of producing comprehensive nationwide data pertaining to the health status, health-related behaviors, and dietary patterns of the Korean population. Initiated in 1998, KNHANES conducts annual cross-sectional studies at a national level. The collected survey data hold substantial importance as empirical evidence in the development and evaluation of health policies, including the National Health Plan. Furthermore, these data are made readily available to researchers, enabling them to engage in further investigations and analyses. The detailed KNHANES has been provided elsewhere [17].

**Figure 1:**
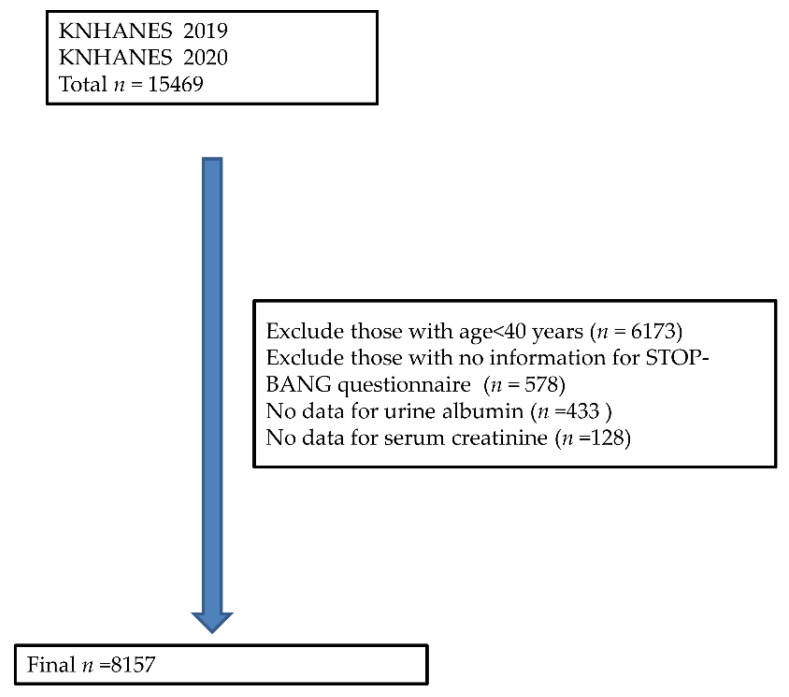
Flow chart of participant exclusion.

After excluding participants below 40 years, those with missing STOP-BANG data, and those without urine albumin and serum creatinine data, the final analysis included 8157 participants. (Figure 1).

### 2.2. STOP-BANG Score

The risk of obstructive sleep apnea was determined using the STOP-BANG, which ranges from 0 to 8. Participants answered eight yes/no questions.

According to the STOP-BANG classification guidelines, the participants were categorized into three groups based on their risk for OSA. These groups were defined as low-risk, intermediate-risk, and high-risk for OSA [18]. A score ranging from 0 to 2 denoted a low level of risk, while a score between 3 and 4 indicated a moderate level of risk. A score between 5 and 8 indicated a high level of risk. Furthermore, participants who answered “yes” to at least two of the four STOP questions and met at least one of the following criteria (being male, having a body mass index (BMI) greater than 35 kg/m^2^, or having a neck circumference greater than 40 cm) were categorized as belonging to the high-risk group.

### 2.3. Assessment of Estimated Glomerular Filtration Rate (eGFR), Albuminuria, and Chronic Kidney Disease

The eGFR was estimated using the 2021 Chronic Kidney Disease Epidemiology Collaboration (CKD-EPI) serum creatinine equation [19]. Serum creatinine levels were measured using fasting blood samples and the Jaffe rate-blanked and compensated method (Hitachi Automatic Analyzer 7600-210, Hitachi High-Technologies, Tokyo, Japan; CREA, Roche Diagnostics, Mannheim, Germany).

Urine albumin was measured using the turbidimetric method (Roche Diagnostics), and urinary creatinine was determined using the rate-blanked and compensated Jaffe method (Wako, Osaka, Japan), both with a Hitachi Automatic Analyzer 7600 (Hitachi High-Technologies). The albumin-to-creatinine ratio (ACR) was used to define albuminuria, with an ACR value greater than 30 mg/g indicating the presence of albuminuria [20]. Furthermore, the CKD status was classified as recommended by “Kidney Disease: Improving Global Outcomes (KDIGO)” and defined as eGFR < 60 mL/min/1.73 m^2^ or ACR ≥ 30 mg/g [20].

### 2.4. Working Hours

The International Labor Organization (ILO) recommends regulating working hours, and many advanced countries consider 40 h per week as the norm for work [21]. The Labor Standards Act of Korea establishes that the legally permitted working hours are 40 h per week and 8 h per day. Participants were requested to provide information on their average weekly working hours, encompassing any additional hours worked beyond the regular schedule as well as any night shifts, in order to assess their overall working hours. The working more than 40 h per week was defined as long working hours.

### 2.5. Covariates

In this study, participants’ education level, smoking status, alcohol consumption, and physical activity were assessed using self-reported questionnaires. The smoking status was categorized into three groups: lifetime nonsmokers, former smokers (individuals who had previously smoked but have quit), and current smokers (individuals who have smoked at least 100 cigarettes in their lifetime and continue to smoke). Alcohol drinkers were defined as individuals who consumed one or more alcoholic beverages per month within the past year. Physical activity was defined as engaging in either moderate-intensity activity for a minimum of 150 min per week, high-intensity activity for a minimum of 75 min per week, or a combination of both moderate and high-intensity physical activities. The homeostatic model of Insulin Resistance (HOMA-IR) was calculated using the following formula: HOMA-IR = (fasting insulin (uIU/mL) × fasting blood glucose (mg/dL))/405. BMI, calculated as weight divided by height in meters squared (kg/m^2^), and Individuals with a BMI of 25 or higher were considered overweight. Hypertension was defined as: (1) ≥130 mmHg systolic or ≥80 mmHg diastolic blood pressure; or (2) use of antihypertensive medication [22]. Cardiovascular disease was defined as a treated myocardial infarction or angina. Hypercholesterolemia was defined as a total cholesterol level of 240 mg/dL or the use of a cholesterol-lowering medication.

### 2.6. Statistical Analyses

The statistical analyses and graphs were performed using Stata version 17 (Stata Corp., College Station, TX, USA) and the R software version 4.2.2 (The Comprehensive R Archive Network: http://cran.r-project.org, accessed on 1 June 2023). The KNHANES Ⅷ (2019–2020) data were combined and used for complex sample analysis with sampling weights to account for the multilevel sampling design.

To compare characteristics between each sex and OSA, one-way ANOVA or the chi-square test was used as appropriate.

The weighted prevalence of albuminuria and CKD according to the risk of OSA and the trend for increasing prevalence were assessed using the Cochran-Armitage test for trend.

Logistic regression models were used to calculate OR and 95% confidence intervals (95% CI) to evaluate the association between OSA and the risk of CKD and albuminuria. In addition, the multivariable model was adjusted for potential confounding factors of well-known risk factors for CKD as follows: age (years, continuous), sex (men, women), educational level (Low, Medium, High), smoking status (never smoked, former smoker, current smoker), alcohol consumption (not a binge drinker, binge drinker), physical activity (inactive, active), overweight (BMI < 25.0 kg/m^2^, BMI ≥ 25.0 kg/m^2^) hypertension (no, yes), HOMA-IR(continuous), hypercholesterolemia (no, yes), and cardiovascular disease (no, yes).

A *p*-value of 0.05 or less was used to determine statistical significance.

## 3. Results

A total of 8157 participants (men: 3575, women: 4582) were included in the analysis. The prevalence of albuminuria was 9.7%, (men: 10.2%, women: 9.2%), and the high risk of CKD was 11.5% (men: 12.2%, women: 10.9%). The risks of obstructive sleep apnea (low-, intermediate-, and high risk) were 61.4%, 23.5%, and 15.2% (men: 38.5%, 31.4%, and 30.1%; women: 83.3%, 15.9%, and 0.8%). OSA diagnosed by a physician was 0.6% overall, 1.0% men and 0.2% women.

Table 1 shows the participants’ general characteristics categorized according to their risk of OSA. Older age, lower education, higher BMI, HOMA-IR, hypercholesterolemia, higher blood pressure, albuminuria, eGFR, more CKD risk, and cardiovascular disease were common in all men and women in the intermediate- or high-risk groups for OSA compared to the lower-risk groups (Table 1). Compared to women, men had a higher proportion of intermediate or high risk of obstructive sleep apnea (OSA) as well as long working hours (Table 1). In men, there is a difference in long working hours based on the risk level of OSA, while no difference was observed in longer working hours among women in the risk group of OSA (Table 1). 

**Figure 2:**
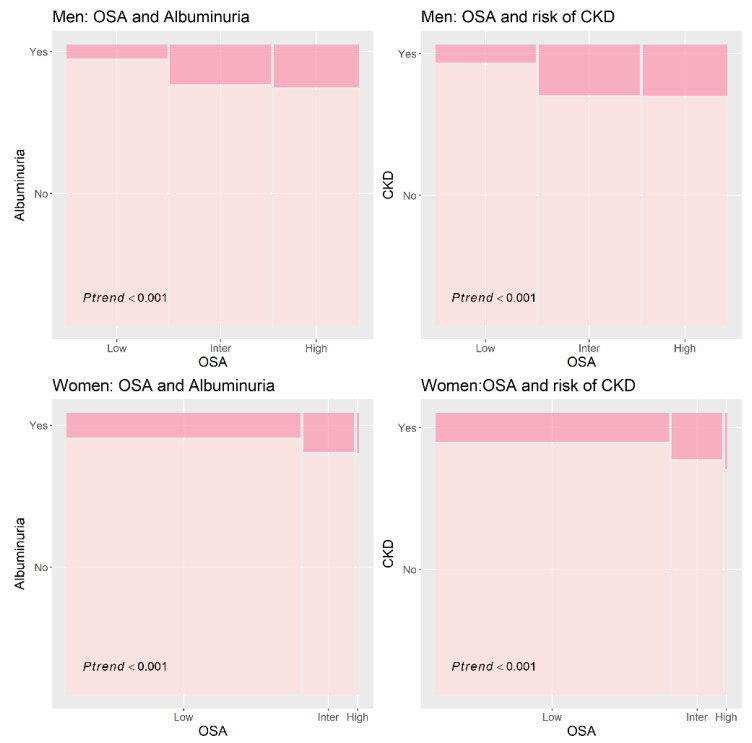
Prevalence of albuminuria and chronic kidney disease according to OSA risk. Abbreviations: CKD, chronic kidney disease; OSA, obstructive sleep apnea.

In men, the estimated prevalence of albuminuria in the intermediate and high-risk groups for OSA was 14.1% and 15.4%, respectively, while the estimated prevalence of CKD in the intermediate and high-risk groups for OSA was 18.0% and 18.3% respectively (Figure 2). In women, the estimated prevalence of albuminuria in the intermediate and high-risk groups for OSA was 13.9% and 14.3%, respectively, while the estimated prevalence of CKD in the intermediate and high-risk groups for OSA was 16.4% and 20.0%, respectively (Figure 2). The prevalence of albuminuria and CKD significantly increased according to OSA risk (Figure 2).

**Figure 3:**
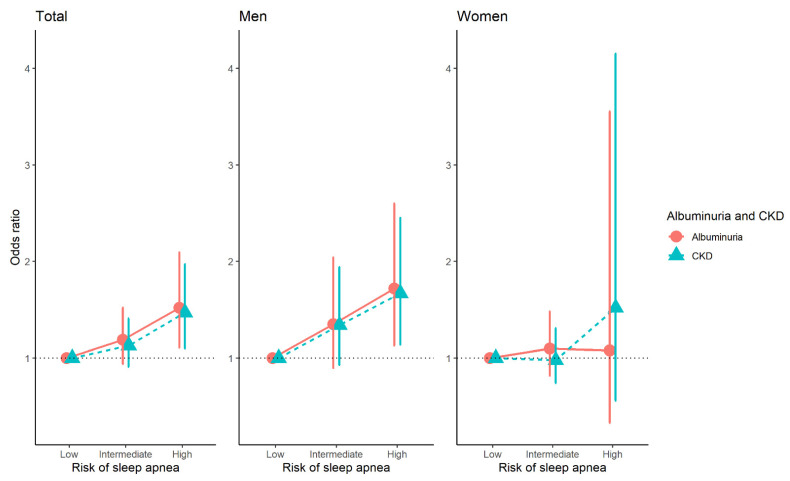
The association between risk of obstructive sleep apnea and albuminuria and chronic kidney disease in sex group using multivariate logistic regression. Abbreviation: CKD, chronic kidney disease. Adjusted for age, sex, educational level, smoking status, alcohol consumption, physical activity, overweight, hypertension, homeostatic model of Insulin Resistance (HOMA-IR), hypercholesterolemia, and cardiovascular disease.

In the fully adjusted model, the OR for albuminuria and CKD risk in the high OSA risk group was 1.52 (95% CI 1.11–2.09) and 1.47 (95% CI 1.1–1.97), respectively (Figure 3). In men, the OR for albuminuria and CKD risk in the high OSA risk group was 1.72 (95% CI 1.13–2.6) and 1.67 (95% CI 1.14–2.45), respectively (Figure 3).

After adjusting for all relevant factors, it was found that compared to the group of individuals with low risk of OSA, the group with high OSA risk had a significantly higher risk of developing albuminuria and chronic kidney disease in men and long working hours (>40 h) (OR 2.09, 95% CI 1.05–4.15; OR 2.04, 95% CI 1.08–3.86, respectively) (Table 2).

## 4. Discussion

The results of this study suggest that individuals in the high-risk OSA group are at a significantly increased risk of developing albuminuria and CKD, particularly among a large population-based sample of men but not women.

In our study, the prevalence of OSA diagnosed by a physician was only 0.6%. However, using the STOP-BAG instrument, the study observed a prevalence of moderate risk, defined by STOP-BAG scores ranging from 3 to 4, to be 23.5% within the population. Additionally, the prevalence of high risk, defined by STOP-BAG scores of 5 or higher, was found to be 15.2% of the population. This finding aligns with a previous United States study [23] that acknowledged the difficulty in determining the prevalence of undiagnosed individuals in the population. Moreover, the study revealed that 44% of the population, as identified by the STOP-BANG score while the current diagnosis rate was only 10% and these results underscore the magnitude of the public health crisis, representing a significant and urgent issue of epidemic proportions [23]. Our previous study demonstrated a positive association between higher scores on the STOP-BAG instrument and an increased risk of multimorbidity [24]. Therefore, specifically targeting individuals with undiagnosed OSA who have high scores on the STOP-BANG instrument for treatment would be of significant importance in public health.

This study found that the prevalence of high-risk OSA was higher in men than in women, which is consistent with the findings of previous studies. OSA is more common in men than in women, with a prevalence ratio of about 3 to 1 and 8:1, respectively [25]. In a study conducted in Germany, the occurrence of OSA was found to be 46% among individuals with an AHI of 5% or higher [26]. Men exhibited a higher prevalence of OSA, with 59% affected, while women had a lower prevalence of 33% [26]. For those with an AHI ≥ 15, the estimated prevalence was 21%, with a higher prevalence observed in men (30%) compared to women (13%) [26].

The mechanisms underlying the association between OSA and CKD are not fully understood. One possible mechanism is that OSA can cause repeated respiratory arrest during sleep, leading to intermittent hypoxia, increased levels of reactive oxygen species and inflammatory mediators, increased systemic inflammation, and endothelial dysfunctions [27]. The renal medulla, in particular, is highly susceptible to hypoxia and the chronic hypoxia hypothesis proposes several mechanisms through which hypoxia contributes to tubulointerstitial injury, which is a precursor to CKD [28]. OSA also causes activation of the sympathetic nervous system and renin-angiotensin-aldosterone system, leading to increased blood pressure and renal vasoconstriction, which can further exacerbate kidney damage [29]. In recent longitudinal study for hypertension patients, individuals with OSA and those in the severe OSA group exhibited a 1.21-fold risk (95% CI: 1.08–1.35) and a 1.27-fold risk (95% CI: 1.09–1.47), respectively, for CKD in the overall population compared to individuals without OSA [30]. Additionally, OSA has been associated with insulin resistance and metabolic dysregulation [31], which may contribute to the development of CKD. OSA can also contribute to an increase in arterial wall stiffness, which, in turn, can harm the kidneys by causing microvascular damage and ischemia in renal tissues [28].

The reasons for sex differences in the association between OSA and the risk of albuminuria and CKD are not entirely clear. One possible explanation is that men may have a higher prevalence and greater severity of OSA than women [32], which may increase their risk of developing albuminuria and CKD. Women are less likely to have supine OSA, and their apnea events are shorter and result in less severe decreases in oxygen saturation [13]. A recent German retrospective study showed that the apnea-hypopnea index was higher in male patients than in female patients [33]. Sleep disordered breathing (SDB) is a condition characterized by an increased resistance to the flow of air in the upper airway, leading to symptoms such as snoring, reduced airflow (hypopnea), and temporary cessation of breathing (apnea) [34]. It has been observed that SDB occurs at higher rates in men compared to women and women with SDB often have different symptoms compared to men, such as daytime fatigue, nightmares, morning headaches, and mood disturbances [34]. The commonly used screening tools may not be as effective in identifying SDB in women, especially those with mild cases. When SDB is categorized based on symptoms, women are more likely to be classified under subtypes characterized by excessive sleepiness and disturbed sleep compared to men [34]. Second, some evidence suggests that sex hormones play an important role in the pathophysiology of albuminuria and CKD in the setting of OSA, particularly in women. Animal studies have shown the potential effects of estrogens on oxidative stress and inflammatory pathways and that endothelial dysfunction may also be affected by sex [34]. Third, factors that may contribute to this difference include the accuracy of OSA measurement by sex. A recent Canadian study suggested that a STOP-BANG score of 5 or higher can accurately identify men with moderate-to-severe OSA, but it does not have diagnostic accuracy for identifying women with OSA [35].

This study also revealed that OSA in men and long working hours (>40 h) were associated with an increased risk of developing albuminuria and CKD. Long working hours may be a potential risk factor for CKD, which is consistent with the results of the previous studies. A recent Korean cohort study that included young-to middle-aged men and women (97856 participants) found a significant association between long working hours and an increased risk of incident CKD [15]. Kim et al. [16] reported that individuals working long hours (41–52 h/week) with diabetes had 1.85 times higher odds of CKD (95% CI 1.15–2.96, *p* = 0.0112) compared to those who worked ≤ 40 h/week or less. As the number of working hours increases, work-related stress can also increase [36], leading to the activation of the systemic inflammatory response, which may play a role in the A development of kidney disease [37]. Another mechanism might be dietary habit change. A recent Korean study explained that long-time workers tend to eat pre-packaged meals or convenience food which, was typically exhibit reduced fiber content while displaying elevated levels of fat, sodium, and overall energy content [38]. In a West Asian study, an increased frequency of fruit consumption demonstrated a negative association with the risk of OSA, whereas a dietary pattern characterized by higher consumption of animal innards, fried food, salted food, carbonated beverages, and non-carbonated beverages was associated with an elevated risk of OSA [39]. A dietary pattern characterized by high levels of dietary fat and sugar was found to be positively correlated with the occurrence of CKD [40].

Our study has several strengths, including the evaluation of the association between OSA and albuminuria and CKD in a nationally representative general population and the use of a simple and easy-to-use STOP-BANG model to define OSA. Sex differences in the association between OSA, albuminuria, and CKD were revealed, and a subgroup analysis of long working hours was performed. To the best of our knowledge, this is the first study to conduct such an analysis.

Our study has limitations. First, with the cross-sectional nature of the study, we could not establish causal relationships. Some articles had indicated the presence of a bidirectional relationship between OSA and CKD and CKD, and may contribute to an elevated vulnerability to sleep apnea, and this association can be attributed to various mechanisms, including uremia-induced neuropathy or myopathy, altered chemosensitivity, and hypervolemia [41]. Second, PSG remains the gold standard for diagnosis. Third, a previous study showed that eating patterns were associated with OSA [39] and CKD [40], however, we do not have eating pattern information. Last we were unable to consider the potential effect of continuous positive airway pressure treatment as the KNHANES did not survey its use.

## 5. Conclusions

The current study presents empirical evidence that supports the link between OSA and the increased likelihood of albuminuria and CKD, particularly among men and individuals who experience excessive work-related demands. These findings indicate that the screening and management of OSA may hold significant value as a preventive approach against the onset and progression of kidney disease, particularly within populations at higher risk. However, additional research is necessary to gain a more comprehensive understanding of the underlying mechanisms driving this association and to explore potential interventions aimed at mitigating the risk of kidney disease in individuals with OSA.

## Figures and Tables

**Table 1:** Participants’ characteristics by risk of obstructive sleep apnea.

Variable	Men (*n* = 3575)				Women (*n* = 4582)				Total (*n* = 8157)			
	Low	Intermediate	High	*p* Value	Low	Intermediate	High	*p* Value	Low	Intermediate	High	*p* Value
n	1254	1260	1061		3740	807	35		4994	2067	1096	
Age, y	56.0 ± 12.1	64.6 ± 10.0	59.1 ± 11.1	**<0.001**	58.9 ± 11.9	65.5 ± 9.0	63.6 ± 9.6	**<0.001**	58.2 ± 12.0	65.0 ± 9.6	59.2 ± 11.1	**<0.001**
Education												
Low	198 (12.2)	401 (28.9)	266 (18.2)	**<0.001**	1229 (29.4)	483 (55.1)	20 (46.2)	**<0.001**	1427 (24.2)	884 (38.5)	286 (19.0)	**<0.001**
Medium	372 (35.4)	392 (36.1)	345 (33.2)		1120 (35.8)	215 (29.5)	10 (36.4)		1492 (35.7)	607 (33.7)	355 (33.3)	
High	569 (52.5)	344 (35.0)	447 (48.6)		1089 (34.8)	107 (15.4)	5 (17.4)		1658 (40.2)	451 (27.8)	452 (47.7)	
Not response	115	123	3		302	2	0		417	125	3	
Smoking												
non smoker	251 (20.5)	254 (19.6)	173 (16.5)	**0.001**	3368 (91.0)	725 (90.1)	33 (94.4)	0.562	3619 (69.3)	979 (44.1)	206 (18.7)	**<0.001**
Former smoker	574 (44.6)	674 (52.6)	555 (50.3)		200 (5.2)	40 (5.0)	2 (5.6)		774 (17.3)	714 (36.1)	557 (49.1)	
Current smoker	418 (34.9)	314 (27.8)	331 (33.2)		135 (3.8)	39 (4.9)	0 (0)		553 (13.3)	353 (19.9)	331 (32.3)	
Not response	11	18	2		37	3	0		48	21	2	
Alcohol consumption												
Non-drinker	436 (33.1)	421 (31.3)	311 (28.3)	0.098	2449 (63.9)	577 (70.7)	26 (75.5)	**0.004**	2885 (54.4)	998 (45.0)	337 (29.6)	**<0.001**
Alcohol drinker	808 (66.9)	824 (68.7)	748 (71.7)		1259 (36.1)	228 (29.3)	9 (24.5)		2067 (45.6)	1052 (55.0)	757 (70.4)	
Not response	10	15	2		32	2	0		42	17	2	
Physical activity												
No	660 (56.5)	652 (56.3)	608 (59.3)	0.378	2116 (61.2)	549 (67.4)	22 (68.6)	**0.027**	2776 (59.7)	1201 (60.3)	630 (59.6)	0.920
Yes	480 (43.5)	484 (43.7)	448 (40.7)		1319 (38.8)	255 (32.6)	13 (31.4)		1799 (40.3)	739 (39.7)	461 (40.4)	
Not response	114	124	5		305	3	0		419	127	5	
BMI												
<25	901 (70.3)	677 (50.2)	481 (43.8)	**<0.001**	2615 (71.0)	409 (50.3)	10 (21.5)	**<0.001**	3516 (70.8)	1086 (50.3)	491 (43.2)	**<0.001**
≥25	353 (29.7)	583 (49.8)	580 (56.2)		1125 (29.0)	398 (49.7)	25 (78.5)		1478 (29.2)	981 (49.7)	605 (56.8)	
HOMA-IR	2.1 ± 2.0	2.7 ± 3.2	3.0 ± 3.4	**<0.001**	2.2 ± 2.1	3.1 ± 4.0	3.9 ± 2.9	**<0.001**	2.2 ± 2.1	2.9 ± 3.5	3.1 ± 3.4	**<0.001**
Hypercholesterolemia	232 (19.3)	384 (32.2)	352 (35.0)	**<0.001**	1183 (30.4)	412 (51.4)	16 (52.3)	**<0.001**	1415 (27.0)	796 (38.8)	368 (35.4)	**<0.001**
Not response					107	3	1		144	62	34	
High blood pressure	543 (44.8)	998 (78.3)	893 (82.7)	**<0.001**	1834 (45.8)	711 (87.5)	35 (100)	**<0.001**	2377 (45.5)	1709 (81.5)	928 (83.2)	**<0.001**
Albuminuria (uACR)												
<30	1191 (94.8)	1082 (87.0)	898 (86.2)	**<0.001**	3417 (92.0)	695 (85.0)	30 (83.7)	**<0.001**	4608 (92.9)	1777 (86.3)	928 (86.1)	**<0.001**
≥30	63 (5.2)	178 (13.0)	163 (13.8)		323 (8.0)	112 (15.0)	5 (16.3)		386 (7.1)	290 (13.7)	168 (13.9)	
eGFR												
<60	1234 (99.1)	1179 (94.9)	1012 (96.5)	**<0.001**	3641 (97.7)	766 (95.2)	33 (91.4)	**0.004**	4875 (98.1)	1945 (95.0)	1045 (96.3)	**<0.001**
≥60	20 (0.9)	81 (5.1)	49 (3.5)		99 (2.3)	41 (4.8)	2 (8.6)		119 (1.9)	122 (5.0)	51 (3.7)	
High risk of CKD												
No	1173 (94.0)	1033 (83.7)	867 (84.0)	**<0.001**	3354 (90.5)	675 (83.0)	28 (75.1)	**<0.001**	4527 (91.5)	1708 (83.5)	895 (83.8)	**<0.001**
Yes	81 (6.0)	227 (16.3)	194 (16.0)		386 (9.5)	132 (17.0)	7 (24.9)		467 (8.5)	359 (16.5)	201 (16.2)	
Cardiovascular disease												
No	1110 (98.0)	1075 (95.4)	1000 (95.4)	**<0.001**	3388 (98.4)	768 (96.0)	34 (97.5)	**<0.001**	4498 (98.3)	1843 (95.6)	1034 (95.5)	**<0.001**
Yes	34 (2.0)	64 (4.6)	59 (4.6)		67 (1.6)	39 (4.0)	1 (2.5)		101 (1.7)	103 (4.4)	60 (4.5)	
Not response	110	121	2		285	0	0		395	121	2	
Working hours												
≤40	787 (58.4)	890 (64.8)	657 (56.6)	**0.003**	3166 (84.3)	700 (86.4)	30 (78.6)	0.423	3953 (76.3)	1059 (72.2)	687 (57.2)	**<0.001**
>40	467 (41.6)	370 (35.2)	404 (43.4)		574 (15.7)	107 (13.6)	5 (21.4)		1041 (23.7)	477 (27.8)	409 (42.8)	

The data were presented as mean ± standard deviation for continuous variables and number (percentage) for categorical variables. Categorical variables were analyzed using the chi-square test, while continuous variables were analyzed using one-way ANOVA. Statistical significance was indicated by bold numbers. Abbreviations: uACR, Urine Albumin-Creatinine Ratio; BMI, body mass index; CKD, chronic kidney disease; eGFR, estimated *Glomerular filtration rate.*

**Table 2:** The association between risk of obstructive sleep apnea and albuminuria and chronic kidney disease in gender group and working hours using multivariate logistic regression.

	OSA	Albuminuria	CKD
		Odds Ratio (95% CI)	Odds Ratio (95% CI)
Men			
Working hours > 40 h	Low	1 (Reference)	1 (Reference)
	Intermediate	1.37 (0.67–2.81)	1.28 (0.68–2.41)
	High	**2.09 (1.05–4.15)**	**2.04 (1.08–3.86)**
Working hours ≤ 40 h	Low	1 (Reference)	1 (Reference)
	Intermediate	1.35 (0.81–2.24)	1.39 (0.88–2.20)
	High	1.54 (0.90–2.64)	1.51 (0.93–2.47)
Women			
Working hours > 40 h	Low	1 (Reference)	1 (Reference)
	Intermediate	1.02 (0.33–3.11)	0.98 (0.32–2.95)
	High	1.17 (0.18–7.80)	6.22 (0.88–44.10)
Working hours ≤ 40 h	Low	1 (Reference)	1 (Reference)
	Intermediate	1.10 (0.81–1.50)	0.97 (0.73–1.30)
	High	0.84 (0.23–3.09)	0.77 (0.25–2.37)

Abbreviations: CI, confidence interval; CKD, chronic kidney disease; OSA, obstructive sleep apnea. Statistical significance was indicated by bold numbers. Adjusted for age, educational level, smoking status, alcohol consumption, physical activity, overweight, hypertension, homeostatic model of Insulin Resistance (HOMA-IR), hypercholesterolemia, and cardiovascular disease.

## Data Availability

The data are freely available on the website (https://knhanes.kdca.go.kr/knhanes/main.do (accessed on 1 June 2023)).
